# Higher Expression Levels of *SSX1* and *SSX2* in Patients with Colon Cancer: Regulated In Vitro by the Inhibition of Methylation and Histone Deacetylation

**DOI:** 10.3390/medicina59050988

**Published:** 2023-05-20

**Authors:** Turki M. Alrubie, Abdullah M. Alamri, Bader O. Almutairi, Abdulwahed F. Alrefaei, Maha M. Arafah, Mohammad Alanazi, Abdelhabib Semlali, Mikhlid H. Almutairi

**Affiliations:** 1 Zoology Department, College of Science, King Saud University, Riyadh 11451, Saudi Arabia; 442106519@student.ksu.edu.sa (T.M.A.); bomotairi@ksu.edu.sa (B.O.A.); afrefaei@ksu.edu.sa (A.F.A.); 2 Genome Research Chair, Department of Biochemistry, College of Science, King Saud University, Riyadh 11495, Saudi Arabia; abdullah@ksu.edu.sa (A.M.A.); msanazi@ksu.edu.sa (M.A.); 3 Pathology Department, College of Medicine, King Saud University, Riyadh 11451, Saudi Arabia; marafah@ksu.edu.sa; 4 Groupe de Recherche en Écologie Buccale, Faculté de Médecine Dentaire, Université Laval, 2420 Rue de la Terrasse, Local 1758, Québec, QC G1V 0A6, Canada; abdelhabib.semlali@greb.ulaval.ca

**Keywords:** *SSX1*, *SSX2*, *SSX3*, 5-aza-2′-deoxycytidine, trichostatin, colon cancer, expression

## Abstract

*Background and Objectives*: Colon cancer (CC) has a high mortality rate and is often diagnosed at an advanced stage in Saudi Arabia. Thus, the identification and characterization of potential new cancer-specific biomarkers are imperative for improving the diagnosis of CC by detecting it at an early stage. Cancer-testis (CT) genes have been identified as potential biomarkers for the early diagnosis of various cancers. Among the CT genes are those belonging to the *SSX* family. In order to assess the usefulness of *SSX* family genes as cancer biomarkers for the detection of early-stage CC, the goal of this research was to validate the expressions of these genes in patients with CC and in matched patients with normal colons (NCs). *Materials and Methods:* RT-PCR assays were used to analyze the *SSX1*, *SSX2*, and *SSX3* family gene expression levels in 30 neighboring NC and CC tissue samples from male Saudi patients. Epigenetic alterations were also tested in vitro using qRT-PCR analysis to determine whether reduced DNA methyltransferase or histone deacetylation could stimulate *SSX* gene expression via 5-aza-2′-deoxycytidine and trichostatin treatments, respectively. *Results:* The RT-PCR results showed *SSX1* and *SSX2* gene expression in 10% and 20% of the CC tissue specimens, respectively, but not in any of the NC tissue specimens. However, no *SSX3* expression was detected in any of the examined CC or NC tissue samples. In addition, the qRT-PCR results showed significantly higher *SSX1* and *SSX2* expression levels in the CC tissue samples than in the NC tissue samples. The 5-aza-2′-deoxycytidine and trichostatin treatments significantly induced the mRNA expression levels of the *SSX1*, *SSX2*, and *SSX3* genes in the CC cells in vitro. *Conclusions:* These findings suggest that *SSX1* and *SSX2* are potentially suitable candidate biomarkers for CC. Their expressions can be regulated via hypomethylating and histone deacetylase treatments, subsequently providing a potential therapeutic target for CC.

## 1. Introduction

Colon cancer (CC) is the third and fourth most common cause of cancer-related death worldwide among males and females, respectively [1]. In Saudi Arabia, it is the leading cause of mortality in both sexes and ranks as the first and third most frequently diagnosed malignancy in men and women, respectively [2]. Furthermore, the prevalence rate of CC is high among Saudi men and women between the ages of 55 and 58 years [3]. However, a recent study indicated that CC has become more prevalent among younger age groups in the Saudi population in recent years [4]. This high mortality and prevalence are due to two main reasons: the limited effectiveness of the available treatments and diagnoses reached when symptoms emerge at advanced stages [5]. Moreover, according to the latest estimates, more than 73% of CC cases are diagnosed at late stages in Saudi patients [6]. Consequently, the identification and characterization of new potential cancer-specific biomarkers have become imperative in improving the diagnosis of CC by detecting it at an early stage [5,7].

Several cancer-testis (CT) genes have been identified as promising biomarkers for the early detection of cancer. In adults, these genes are expressed specifically in the germline cells of the testis. However, they might be aberrantly activated in some types of cancer, including melanoma, ovarian cancer [8], and CC [9]. In testicular tissue, large percentages of CT genes are involved in testicular development, spermatogenesis, and fertilization; however, the functions of most of these genes are still unclear [7,10]. In addition, CT genes may be expressed in cancer cells because of the similarities between testicular and tumor cells, including cell division, immigration, and immortalization [10]. Therefore, the specificity and distinctive expressions of CT genes in normal testicular and cancer cells make them potential biomarkers for cancer diagnosis and immunotherapeutic targets in cancers, including CC [9,11].

To our knowledge, approximately 70 CT gene families and more than 170 members have been identified by the Ludwig Institute for Cancer Research and are listed on a free website (http://www.cta.lncc.br/ accessed 1 October 2022) [12]. The synovial sarcoma X chromosome breakpoint (*SSX*) family is one such CT group and consists of nine homologous genes numbered from *SSX1* to *SSX9* [13,14]. A previous study indicated that *SSX1*, *SSX2*, *SSX3*, *SSX4*, *SSX5*, and *SSX6* were completely inactive in normal tissues except in the testis, whereas *SSX7*, *SSX8*, and *SSX9* were inactive in all normal tissues, including the testis [13]. Moreover, Gure et al. (1997) and Türeci et al. (1998) observed that the family members *SSX1*, *SSX2*, *SSX4*, and *SSX5* were expressed in normal tissues only in the testis and in a wide variety of cancers, including melanoma, lymphoma, and head and neck, ovarian, colorectal, and breast cancers, whereas *SSX3* was expressed in normal testicular tissues but not in cancer tissues [15,16]. Furthermore, a recent study reported that *SSX2* expression could be used as a biomarker for the early diagnosis of CC owing to its high expression level in CC tissues when compared to normal tissues [17].

DNA methylation and histone acetylation are epigenetic processes that have been shown to influence the expressions of various CT genes [7,9,11,18]. With regard to DNA methylation, DNA methyltransferase enzymes (DNMTs) control the expressions of diverse CT genes by adding large amounts of a methyl group (CH₃) to their promoter regions, silencing them. This process is called DNA hypermethylation [11,18]. By contrast, the treatment of cell lines with DNA methyltransferase inhibitor (DNMTi) drugs, such as 5-aza-2′-deoxycytidine (5-aza-CdR), allows for the experimental DNA hypomethylation of several CT genes, which removes the CH₃ groups and promotes increased CT gene expression levels [7,9]. For example, Almutairi et al. (2022c) demonstrated that the treatment of CC cell lines with 5-aza-CdR correlated with the activation of CT genes, such as *MAGE-A4*, *MAGE-B1*, *SCP2D1*, and *CTAG1A* [7].

Regarding histone acetylation, several CT genes can also be regulated by adding an acetyl group (−COCH₃) via histone acetyltransferases (HATs) and by removing −COCH₃ via histone deacetylases (HDACs), which results in chromatin remodeling through hyperacetylation and hypoacetylation, respectively [11,19]. Moreover, when CC cell lines were experimentally treated with HDAC inhibitor (HDACi) drugs, such as trichostatin A (TSA), several CT genes were activated, including *MAGE-A4*, *MAGE-B1*, *SCP2D1*, *CTAG1A*, *TKTL2*, and *ACTRT1* [7].

The primary objectives of this research were (1) to examine the expressions of *SSX1*, *SSX2*, and *SSX3* in Saudi Arabian patients diagnosed with CC to identify a novel CT gene that has potential usefulness as a biomarker of CC and (2) to evaluate the effects of epigenetic agents on the control of the expressions of these genes. The *SSX* family genes were selected for the following reasons: the *SSX2* gene was chosen on the basis of its expression in CC tissues or cell lines [16,17] and its association with other cancers [8,20]. From the CTA database (http://www.cta.lncc.br/index.php, accessed 1 October 2022), *SSX1* and *SSX3* were randomly selected. In order to examine the specificity of possible CC biomarkers, we used RT-PCR assays to analyze the mRNA expressions of *SSX* genes in CC tissues but not in matched normal colon (NC) tissues in breast and leukemia cancers.

## 2. Materials and Methods

### 2.1. Ethical Approval and Sample Collection

The institutional review board (approval No. HAPO-01-R-011; project No. 56-2020) of Al-Imam Muhammad Ibn Saud Islamic University authorized this research. Participants were recruited from King Khalid University Hospital in Riyadh, Saudi Arabia. All participants included in this study had not received any treatment, including chemotherapy and/or physiotherapy. Clinical examination, endoscopy, imaging, and histological study are standard methods for diagnosing adenocarcinoma. In this study, these methods were used to monitor and diagnose the patients. Furthermore, all participants agreed and signed a written informed consent form for participation and were provided a privacy statement describing their personal data protection. Moreover, all participants were summoned to fill out a self-completed questionnaire, including information on age, family history, personal medical history, allergy symptoms or diseases, and social behaviors such as cigarette smoking and alcohol consumption.

A total of 35 matched CC and NC tissue samples from the same patient were collected in the study, including 30 and 5 samples taken from male and female Saudi patients with CC, respectively. Moreover, 15 samples were taken from female Saudi patients with breast cancer (BC). Furthermore, 12 samples were taken from male Saudi patients with chronic lymphoblastic leukemia (CLL) and compared with 12 normal blood (NB) samples from healthy Saudi men. Collecting fresh CC samples, along with matching NC tissues and BC samples, was done in sterile tubes with *RNAlater* stabilization solution (76106; Thermo Fisher Scientific, Foster City, CA, USA) to preserve and stabilize RNA. However, CLL samples and NB samples were collected into Blood RNA Tube (4342792; Applied Biosystems, Waltham, MA, USA). After that, all the tubes were kept overnight at 4 °C and then transferred into a −80 °C freezer until use.

### 2.2. Sources and Cultures of Human CC Cell Lines and Their Treatments with Epigenetic Drugs (5-aza-CdR or TSA)

In this study, we used HCT116 and Caco-2 human CC cell lines obtained from the chairperson Genome Research Chair (King Saud University, Riyadh, Saudi Arabia). The two cell types were grown in a 5% CO_2_ humidified 37 °C incubator with DMEM (61965026; Thermo Fisher Scientific) with 10% fetal bovine serum (A3160801; Thermo Fisher Scientific).

Dimethyl sulfoxide (DMSO; D8418; Sigma, Hilden, Germany) was used to dissolve and dilute 5-aza-2′-CdR (A3656; Sigma) or TSA (T1952; Sigma) to the final concentration required in this study. Each type of cell line, either HCT116 or Caco-2, was subcultured into four sets. The first set was treated with 10 μM of 5-aza-CdR for 72 h, the second set with DMSO for 72 h (as a negative control for 5-aza-CdR), the third set with 100 nM of TSA for 48 h; and the fourth set, with DMSO for 48 h (as a negative control for TSA). However, the medium containing 5-aza-CdR, TSA, or DMSO was changed every 24 h. The times and concentrations were determined on the basis of the results of our recent publication [7].

### 2.3. RNA Isolation from NC, CC, BC, CLL, NB, and Cultured Cells

According to the recommendations of the manufacturer of the All-Prep DNA/RNA Mini Kit (80204; Qiagen, Hilden, Germany), approximately 30 mg each of the CC, NC, and BC samples was used separately in clean Eppendorf tubes to isolate and purify total RNA. Total RNA was obtained from around 5 × 10⁶ grown cells using the manufacturer-recommended protocol from the All-Prep DNA/RNA Mini Kit. For the NB and CLL samples, the QIAamp RNA Blood Mini Kit (52304; Qiagen) was used to isolate and purify total RNA from 1.5 mL of the whole blood sample in accordance with the manufacturer’s recommendations. Methods indicated in our prior research were used to determine the extracted RNA concentrations [7,9].

### 2.4. Synthesis of cDNA

A high-capacity cDNA reverse transcription kit (4368814; Applied Biosystems, Waltham, MA, USA) was used to convert 2000 ng/µL RNA from each sample into complementary DNA (cDNA) in accordance with the manufacturer’s instructions. After that, the cDNA was diluted at 1:10 and kept at 20 °C.

### 2.5. Design of RT-PCR Primers, RT-PCR Conditions, and Agarose Gel Electrophoresis of RT-PCR Products

All RT-PCR primers were designed using previously described manual and software methods [7,9]. All primers used in this study were supplied by Macrogen Inc. (Seoul, South Korea). Nuclease-free water (129115; Qiagen) was used to dilute the primers to a final concentration of 10 μM (10 pmol/L). Table 1 lists the gene sequences and expected sizes of the RT-PCR products generated from those sequences. To compare the qualities of the normal, cancer, treated, and untreated cDNA samples, we amplified the housekeeping gene *ACTB* as a positive control. Furthermore, the effectiveness of the primer set for each gene was verified using cDNA from human testis total RNA (AM7972; Thermo Fisher Scientific).

For the RT-PCR reaction preparation, 20 µL of the reaction mixture was placed in a clean PCR tube containing 10 µL of BioMix Red (BIO-25006; BioLine, London, UK), 8.4 µL of nuclease-free water, 0.8 µL of diluted cDNA (200 ng/µL), and 0.8 µL of both forward and reverse primers (10 μM) for each gene. The cycling parameters for the RT-PCR protocol were as follows: 5 min at 96 °C (one cycle), followed by 30 s at 96 °C, 30 s at 58 °C, and 30 s at 72 °C (35 cycles), and finally, 5 min of incubation at 72 °C (one cycle).

For gel electrophoresis, 1.5% agarose gel (A9539; Sigma-Aldrich, St. Louis, MO, USA) mixed with ethidium bromide (46067; Sigma) in 1× TBE buffer was used to separate 8 μL of each PCR product with a voltage of 100 for 1 h. In addition, 3 µL of a 100-bp DNA marker (N0467; New England BioLabs, London, UK) was loaded into agarose gels to confirm the sizes of the PCR products.

### 2.6. Design of qRT-PCR Primers and qRT-PCR Setups

Each set of qRT-PCR primers was manually designed using the optimal criteria provided in previous studies [7,9]. All primers were commercially synthesized using Macrogen. Stock primers were diluted with nuclease-free water to achieve their final concentration of 10 μM. The sequences of the qRT-PCR primers and their expected amplicon sizes are displayed in Table 2.

For qRT-PCR reaction preparation, a 96-well plate was used in accordance with the iTaq Universal SYBR Green Supermix (1725120; Bio-Rad, Hercules, CA, USA) instructions. In order to obtain 10 μL of the total amount for each reaction, 5 μL of SYBR Green, 2 μL of diluted cDNA (200 ng/µL), 0.5 μL from both forward and reverse primers (10 μM), and 2.5 μL of nuclease-free water were added to each well. Each sample was duplicated twice using the QuantStudioTM 7 Flex Real-Time PCR System. The qRT-PCR cycling conditions were as follows: initial denaturation at 95 °C for 30 s and then 40 qRT-PCR cycles at 95 °C for 30 s, 58 °C for 30 s, and 72 °C for 30 s. A melting curve analysis was performed upon completion of the 40 cycles. The *GAPDH* housekeeping gene was used to standardize the qRT-PCR results.

### 2.7. Statistical Analysis

Significant differences between the two categories (before and after 5-aza-CdR or TSA treatment) for each gene were analyzed using the SPSS software (ver.22; SPSS Inc., Chicago, IL, USA). In this study, all *p* values within the following ranges were regarded as statistically significant: * *p* ≤ 0.05, ** *p* ≤ 0.01, and *** *p* ≤ 0.001.

### 2.8. In Silico Analysis

By using GeneMANIA tools (University of Toronto, Toronto, ON, Canada), the gene-gene interaction network of the *SSX* genes and their functional associations were created for a network analysis of common genes and the prediction of related genes [21].

### 2.9. The Cancer Genome Atlas (TCGA) Database Analysis

By using the TCGA database, *SSX1*, *SSX2*, and *SSX3* expression levels were examined in different colon adenocarcinoma (COAD) tissue samples and compared with their expression levels in NC tissue samples. The expression patterns of the *SSX1*, *SSX2*, and *SSX3* genes were validated in the COAD and NC tissue samples from the TCGA using datasets provided in OncoDB that were primarily from TCGA and included RNA-seq and clinical data from more than 9000 patients with cancer. For these analyses, RNA-seq data were obtained from the backend database and separated into two groups: the COAD and NC tissue samples. Whether a gene was upregulated or downregulated in the tumor samples was determined by calculating the log2-fold change value between the two groups. The Student *t*-test was used for the differential expression analysis. *p* values ≤ 0.05 were regarded as statistically significant.

## 3. Results

### 3.1. Clinical Parameters of the Study Participants

As CC is more difficult to treat during later stages, late diagnosis is one of the most important causes of increased mortality in Saudi Arabia. Therefore, examining *SSX* family gene expressions (i.e., cancer biomarkers) in a large number of patients with CC should provide insights that will aid in the early diagnosis of malignancy and, thus, increase the likelihood of successful therapy. Table 3 displays the study participants’ baseline clinical characteristics. A total of 74 participants were evaluated, including 35 with NC or CC, 15 with BC, 12 with leukemia, and 12 with normal blood (NB). The mean ages of the patients with CC and BC were 61 years (range, 24–96 years) and 52 years (range, 32–74 years), respectively. The mean ages of the patients with leukemia and the controls with NB were 49 years (range: 39–64 years) and 43 years (range: 33–52 years), respectively. Forty-three percent of the patients with CC were younger than 61 years, while 57% were older than 61 years. Overall, 60% of those with BC, 50% of those with leukemia, and 67% of those with NB were younger than 52, 49, and 43 years, respectively, whereas 40%, 50%, and 33% were older than 52, 49, and 43 years, respectively. The other clinical parameters of the participants are listed in Table 3.

### 3.2. Expression Profiles of the SSX1, SSX2, and SSX3 Genes in the Matched CC and NC Tissues from the Male and Female Patients

**Figure 1:**
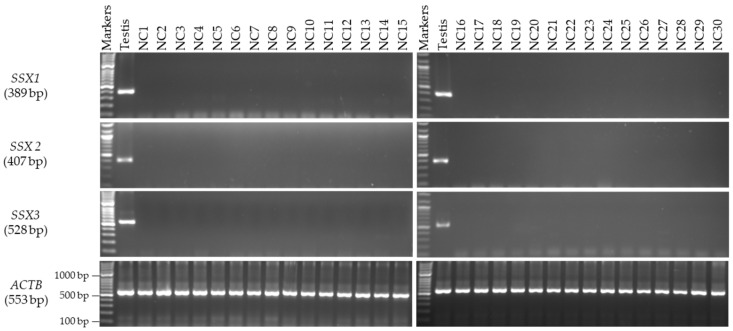
**Detection of *SSX* gene transcripts in the NC tissue samples from male patients.** The agarose gel images display the RT-PCR analysis results for *SSX1*, *SSX2*, and *SSX3*. cDNAs were synthesized from the total RNA from 30 NC tissue samples. The cDNA samples were run with *ACTB* expression as the positive control, and, as predicted, a band of 553 bp was obtained. Each set of primers for a given gene was examined using human testis cDNA. The official names and expected product sizes of the individual genes are presented to the left of the agarose gel images. Abbreviations: NC: normal colon; bp: base pair.

**Figure 2:**
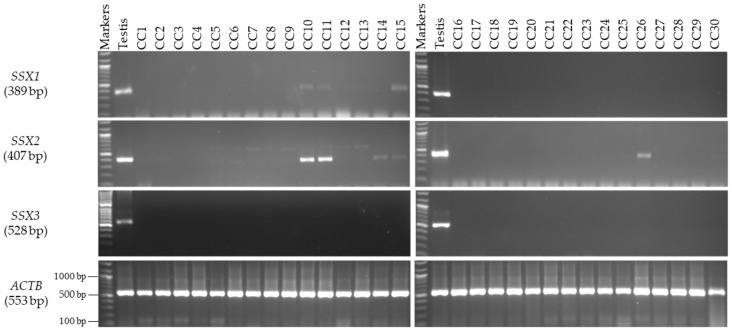
**Detection of *SSX* gene transcripts in the CC tissue samples from male patients.** The agarose gel images display the RT-PCR analysis results for *SSX1*, *SSX2*, and *SSX3*. cDNAs were synthesized from the total RNA from 30 CC tissue samples. The cDNA samples were run with *ACTB* expression as the positive control, and, as predicted, a band of 553 bp was obtained. Each set of primers for a given gene was examined using human testis cDNA. The official names and expected product sizes of the individual genes are presented to the left of the agarose gel images. Abbreviations: CC: colon cancer; bp: base pair.

**Figure 3:**
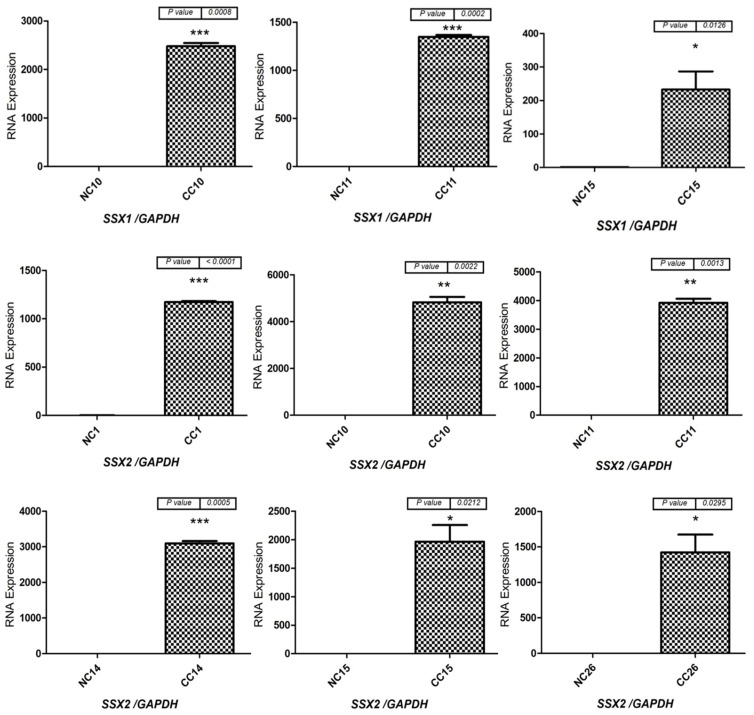
**qRT-PCR results of the positive expressions of *SSX1* and *SSX2* in the NC and CC tissue samples**. In accordance with the RT-PCR results, qRT-PCR analysis was performed to measure the mRNA expressions of *SSX1* and *SSX2* in three and six positive CC tissue samples, respectively, in comparison to those in the matched NC tissue samples. *GAPDH* mRNA was used as a reference to normalize the expression levels. The standard error of the mean for three independent experiments is represented by the error bars for each gene in each CC and NC sample. * *p* ≤ 0.05; ** *p* ≤ 0.01; *** *p* ≤ 0.001. Abbreviations: NC: normal colon; CC: colon cancer; qRT-PCR: quantitative reverse transcription polymerase chain reaction.

The mRNA expression levels of the *SSX* family members were analyzed by first identifying the primers and annealing temperatures that would result in specific product amplification for each member of the *SSX* family. In the male patients, the mRNA levels of the *SSX1*, *SSX2*, and *SSX3* genes were first validated using RT-PCR analysis with various RNAs isolated from 30 human NC tissue samples from Saudi men for the evaluation of testis specificity (Figure 1). The primer of each gene was verified by testing it on cDNA extracted from human testis RNA. The integrity of the cDNAs from the NC and CC samples was validated on the basis of *ACTB* gene expression. By using RT-PCR analysis, *SSX1* and *SSX2* were found to be expressed in 10% and 20% of the CC tissue samples, respectively (Figure 2), but were not detected in any of the NC tissue specimens (Figure 1). However, no detectable *SSX3* expression was found in any of the examined CC (Figure 2) or NC tissue samples (Figure 1). For further analysis, the target samples for RT-PCR were tested by using qRT-PCR for *SSX1* in three samples and *SSX2* in six samples of CC compared to their normal matching tissues. The rest of the CC and NC samples were not analyzed with qRT-PCR due to the absence of detectable expressions of *SSX1* and *SSX2* in agarose gel (Figure 3). The expression level of each gene was validated in the NC and CC tissues from the same sample. The expression level of each gene in the NC tissues was normalized to *GAPDH* and compared with that in the corresponding CC tissues. Figure 3 presents the qRT-PCR results, demonstrating significantly higher *SSX1* and *SSX2* expression levels in the CC tissues than in the NC tissues. Thus, the RT-PCR findings matched the qRT-PCR results. The expression of the level of *SSX3* was not analyzed using qRT-PCR because of the absence of detectable expression levels in the agarose gel in the NC and CC tissues. Moreover, in this study, *SSX1* and *SSX2* gene expressions were considered positive when a band was found in the NC and CC tissue samples. However, only *SSX2* showed statistically significant positive expression in the CC tissue samples relative to the NC tissue samples (*p* = 0.009; Table 4).

**Figure 4:**
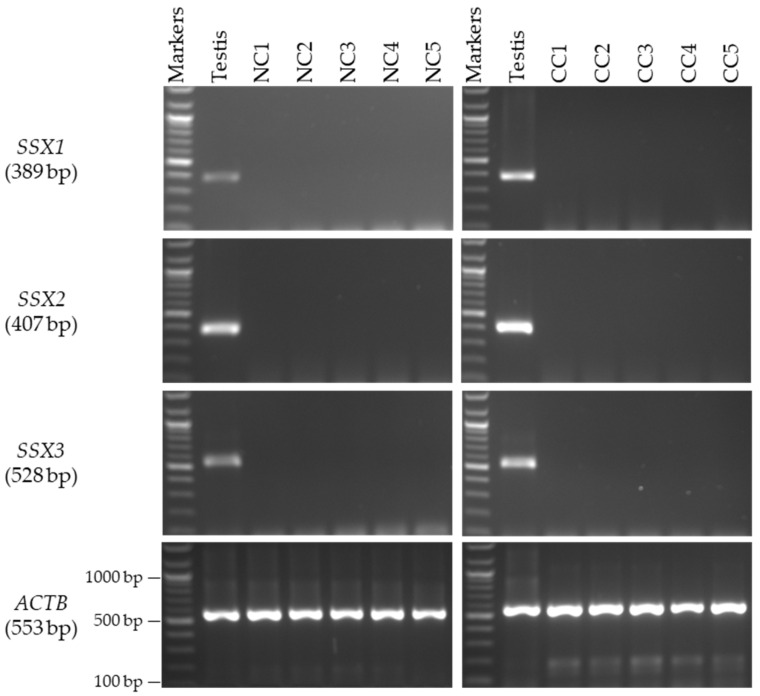
**Detection of *SSX* gene transcripts in the NC and CC tissue samples from female patients.** The agarose gel images display the RT-PCR analysis results for *SSX1*, *SSX2*, and *SSX3*. The cDNAs were synthesized from the total RNA from five NC and CC tissue samples. The cDNA samples were run with *ACTB* expression as the positive control, and as predicted, a band of 553 bp was obtained. Each set of primers for a given gene was examined using human testis cDNA. The official names and expected product sizes of the individual genes are presented to the left of the agarose gel images. Abbreviations: NC: normal colon; CC: colon cancer; bp: base pair.

In the female patients, the mRNA levels of the *SSX1*, *SSX2*, and *SSX3* genes were validated using a panel of RNAs obtained from the NC tissue samples from five female Saudi patients to determine the testis specificity of the mRNAs. No detectable expressions of the *SSX1*, *SSX2*, and *SSX3* genes were found in any of the examined NC or CC tissue samples from the female patients (Figure 4).

### 3.3. Screening of the SSX Genes in CLL and BC Tissue Samples

**Figure 5:**
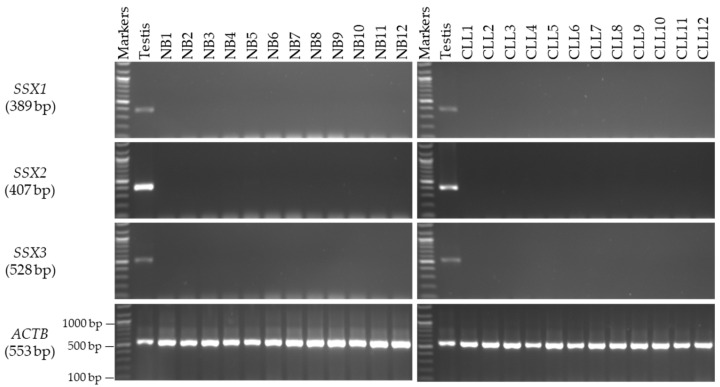
**Detection of *SSX* gene transcripts in male CLL and NB tissue samples.** The agarose gel images display the RT-PCR analysis results for *SSX1*, *SSX2*, and *SSX3*. The cDNAs were synthesized from the total RNA from 12 CLL and 12 NB tissue samples. The cDNA samples were run with *ACTB* expression as the positive control, and, as predicted, a band of 553 bp was obtained. Each set of primers for a given gene was examined using human testis cDNA. The official names and expected product sizes of the individual genes are presented to the left of the agarose gel images. Abbreviations: CLL: chronic lymphoblastic leukemia; NB: normal blood; bp: base pair.

**Figure 6:**
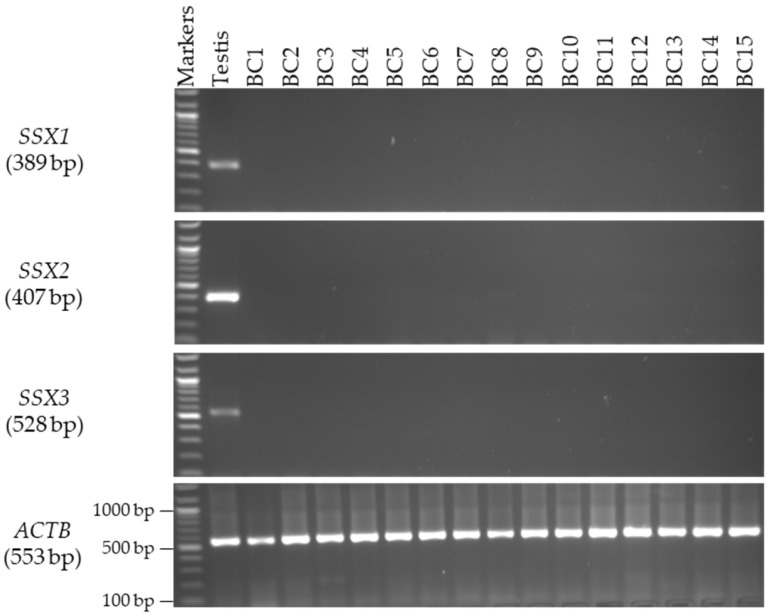
**Detection of *SSX* gene transcripts in female BC tissue samples.** The agarose gel images display the RT-PCR analysis results for *SSX1*, *SSX2*, and *SSX3*. The cDNA samples were run with *ACTB* expression as the positive control, and, as predicted, a band of 553 bp was obtained. Each set of primers for a given gene was examined using human testis cDNA. The official names and expected product sizes of the individual genes are presented to the left of the agarose gel images. Abbreviations: BC: breast cancer; bp: base pair.

This screening was conducted to determine the specificity of *SSX1*, *SSX2*, and *SSX3* in additional cancer tissue samples, including leukemia in males and BC in females. The RT-PCR screening results showed that none of the *SSX1*, *SSX2*, and *SSX3* genes were expressed in either of the CLL tissue samples (Figure 5, right) when compared to the NB samples (Figure 5, left) or BC tissue samples (Figure 6).

### 3.4. Effects of 5-aza-2′-CdR and TSA on SSX Gene Expressions in CC Cell Lines

Hypomethylating agents, such as 5-aza-2′-CdR, or histone deacetylase inhibitors, such as TSA, can increase the expression levels of multiple CT genes [7,11]. Most of these genes are X-CT genes, the silencing of which requires the hypermethylation of DNA sequences. Therefore, we questioned whether the expressions of *SSX1*, *SSX2*, and *SSX3* could be regulated via treatment with 5-aza-2′-CdR or TSA agents and whether the expressions of some *SSX* genes in CC tissue samples might be affected by altered methylation and histone deacetylation mechanisms. We found no change in the morphology of the tumor cells treated with the 5-aza-2′-CdR or TSA agents. The mRNA level of each gene was measured in cells treated with 5-aza-CdR or TSA as compared to the cells treated with DMSO. DMSO was used as the solvent for both treatment drugs; therefore, 10 µL of DMSO was added to the cells in both groups as a control to determine its effects on gene expression.

In order to examine whether reduced DNA methyltransferase activity can activate *SSX1*, *SSX2*, and *SSX3* gene expression, the HCT116 and Caco-2 cell lines were treated with 10 µM 5-aza-2′-CdR for 72 h. Then, the cDNA was synthesized, and qRT-PCR was performed, as described in Section 2.4 and Section 2.6.

**Figure 7:**
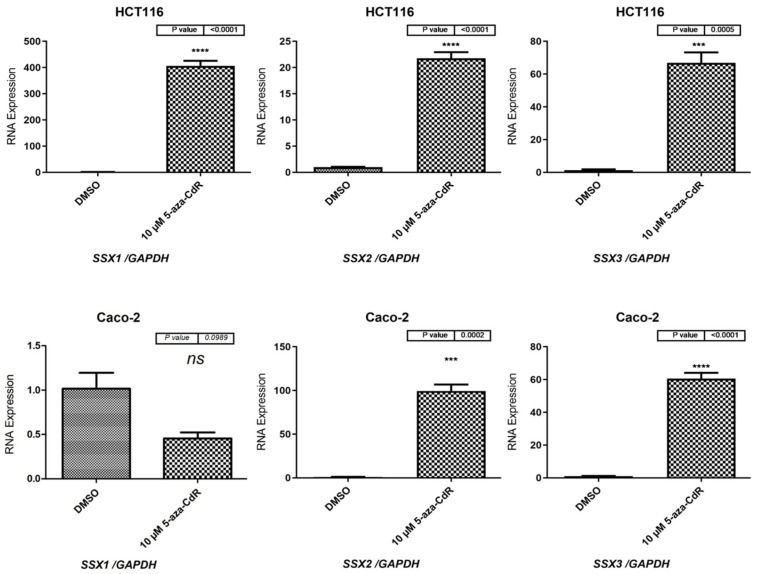
qRT-PCR results for *SSX1*, *SSX2*, and *SSX3* in HCT116 and Caco-2 cells after treatment with 10 µM of 5-aza-2′-CdR for 72 h. The bar graphs show the *SSX1*, *SSX2*, and *SSX3* expression levels in the HCT116 and Caco-2 cells before and after treatment with 5-aza-2′-CdR. DMSO was also utilized as a solvent for the 5-aza-2′-CdR solution and was applied to the control HCT116 and Caco-2 cells. *GAPDH* mRNA was used as a reference to normalize the gene expression levels. The standard error of the mean for three independent experiments is represented by the error bars. *** *p* ≤ 0.001; **** *p* ≤ 0.0001. Abbreviation: qRT-PCR: quantitative reverse transcription polymerase chain reaction; ns: not-significant.

The qRT-PCR results for the HCT116 cells indicated that the mRNA expression levels of the *SSX1*, *SSX2*, and *SSX3* genes were significantly induced in the cells treated with 5-aza-CdR when compared with those treated with DMSO (*p* < 0.0001; *p* < 0.0001; and *p* = 0.0005, respectively: Figure 7). In addition, the mRNA expressions of the *SSX1* and *SSX2* genes were more activated than those of the *SSX3* gene. The qRT-PCR results showed that *SSX2* and *SSX3* expression was significantly induced in the Caco-2 cells treated with 5-aza-CdR (*p* = 0.0002 and *p* < 0.0001, respectively: Figure 7). However, the *SSX1* gene did not exhibit statistically significant changes in the Caco-2 cells treated with 5-aza-CdR when compared to those treated with DMSO, as shown in Figure 7.

**Figure 8:**
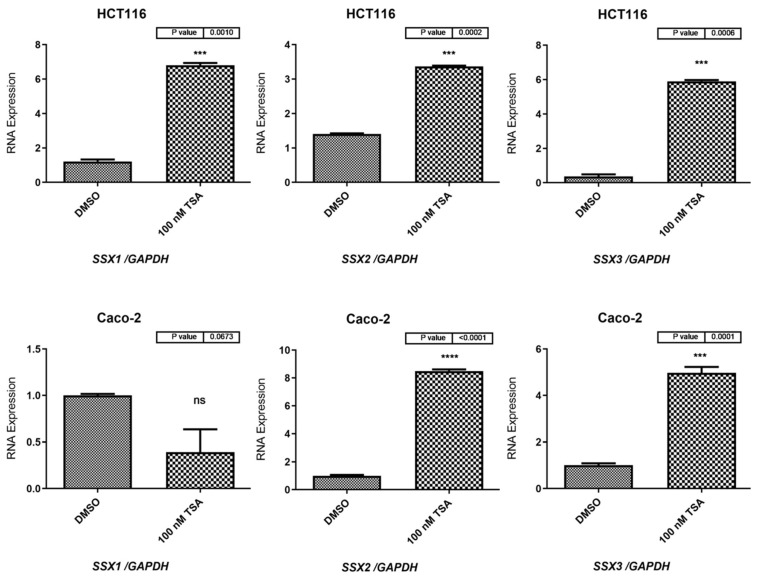
**qRT-PCR analyses for *SSX1*, *SSX2*, and *SSX3* in HCT116 and Caco-2 cells after treatment with 100 nM of TSA for 48 h.** The bar graphs show the *SSX1*, *SSX2*, and *SSX3* expression levels in the HCT116 and Caco-2 cells before and after treatment with TSA. DMSO was also utilized as a solvent for the TSA solution and was applied to the control HCT116 and Caco-2 cells. *GAPDH* mRNA was used as a reference to normalize the gene expression levels. The standard error of the mean for three independent experiments is represented by the error bars. *** *p* ≤ 0.001; **** *p* ≤ 0.0001. Abbreviation: qRT-PCR: quantitative reverse transcription polymerase chain reaction; ns: not-significant.

Next, the significance of histone deacetylation in the repression of *SSX* family genes was investigated by treating HCT116 and Caco-2 cells with 100 nM of TSA for 48 h. The mRNA expressions of the *SSX1*, *SSX2*, and *SSX3* genes in the HCT116 cells significantly increased when treated with the TSA agent (*p* = 0.0010; *p* = 0.0002; *p* = 0.0006, respectively: Figure 8). The qRT-PCR results showed that *SSX2* and *SSX3* gene expression significantly increased in the Caco-2 cells treated with TSA (*p* < 0.0001 and *p* = 0.0001, respectively: Figure 8). However, the *SSX1* gene did not exhibit statistically significant changes in the Caco-2 cells treated with TSA when compared with those treated with DMSO, as shown in Figure 8.

### 3.5. Gene–Gene Interaction Network

**Figure 9:**
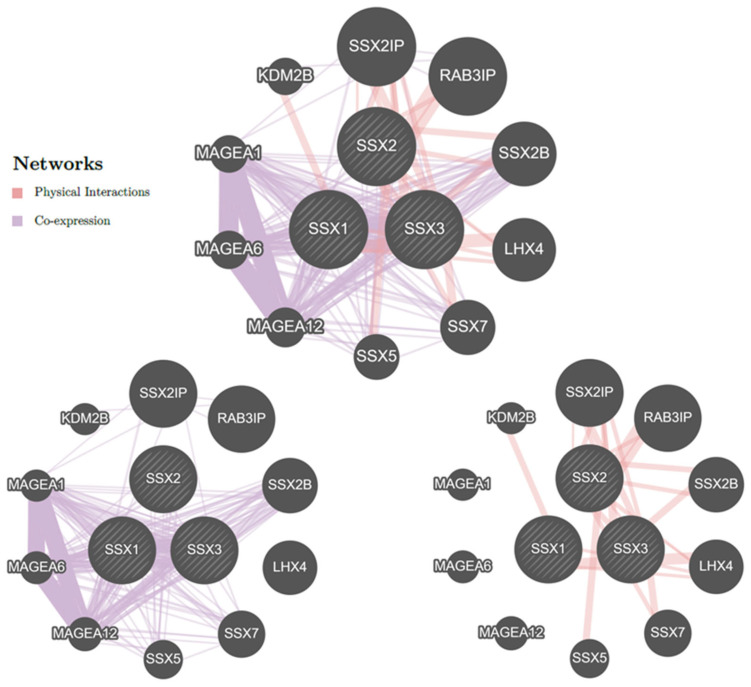
**The gene–gene interaction network for *SSX1*, *SSX2*, and *SSX3* members using the GeneMANIA database.** Circular *SSX* genes are represented by the center nodes. The top 10 genes most commonly found in close proximity to *SSX* genes are shown. The lines indicate additional genes, and the edges illustrate their interactions with *SSX* genes.

The default setting of GeneMANIA was used to build a gene–gene interaction network for analyzing the *SSX* gene functions. The core node represented the *SSX* gene members that were surrounded by 10 nodes, defining the other genes that were strongly connected to the *SSX* genes in terms of both co-expression and physical interactions (top of Figure 9). The *SSX1*, *SSX2*, and *SSX3* genes were highlighted as being co-expressed with 10 other genes in the following ranking: *SSX2IP*, *RAB3IP*, *SSX2B*, *LHX4*, *SSX7*, *SSX5*, *MAGEA12*, *MAGEA6*, *MAGEA1*, and *KDM2B* (colored purple in the bottom left of Figure 9). However, the GeneMANIA program revealed that the interconnected network of the *SSX2IP*, *RAB3IP*, *SSX2B*, *LHX4*, *SSX7*, *SSX5*, and *KDM2B* genes had real physical interactions (colored pink in the bottom right of Figure 9). Furthermore, the analysis revealed that the co-expressions and physical interactions accounted for 18.53% and 81.47%, respectively.

## 4. Discussion

CT antigens are prospective cancer-specific biomarkers with potential diagnostic, prognostic, or therapeutic uses. The current classification approach for CT genes was developed by Hoffman et al. [8]. On the basis of an in silico pipeline, a subgroup of human meiotic genes was described as being composed of CT genes and presented a highly restricted cancer-specific marker [20,22].

Primers that specifically amplify individual *SSX* family members were identified in this study. These primers were used to validate *SSX1*, *SSX2*, and *SSX3* expression in CC by using RT-PCR on fresh tissue samples from 35 patients with CC and the corresponding NC tissues. The primers selected from different exons were designed to avoid false-positive outcomes resulting from contaminated genomic DNA (as shown in Table 1). After validation, the RT-PCR screening identified the *SSX2* gene as a potential novel CT-restricted gene, possibly representing an optimal candidate CC biomarker because it was expressed in 20% (*p* = 0.009) of the CC tissue samples, respectively, but not in the NC tissues. The activation of CT genes in cancer is likely associated with demethylation or histone deacetylation inhibition [7,11]. The RT-PCR results showed *SSX1* gene expression in 10% (*p* = 0.078) of the CC tissue specimens but not in any of the NC tissue specimens. For the *SSX1* and *SSX2* genes, the qRT-PCR findings were consistent with the RT-PCR results, demonstrating that these genes are expressed only in CC tissues and not in NC tissues. In order to determine the CC specificity of the *SSX1* and *SSX2* genes, BC, CLL, and NB tissue samples were examined; however, neither gene was expressed in any of the tissue samples.

The expressions of the *SSX1* and *SSX2* genes were found in the advanced grades of CC tissue samples, according to the clinical data of the study participants (grades II and III). Consistent with previous reports on *SSX* family expression in a range of human cancers (9, 17, 18), *SSX* gene expression has been correlated with more advanced stages of disease [23,24,25]. Previous studies have identified the same findings for *SSX* genes in patients with CC [17]. *SSX* genes were expressed in 32.4% of CC tissue samples but were not detected in NC tissue samples [17]. A previous study showed that *SSX2* was expressed in prostate cell lines, but *SSX1* was not expressed in the same prostate cell lines [26]. High *SSX1* and *SSX2* expression levels were observed in patients with hepatocellular carcinoma, which suggests that they might be used as a cancer marker [27,28]. These inconsistent results could be due to the differences in the primer sets used or in the physiology of the clinical samples. Our study is the first to validate *SSX* gene expressions in Saudi patients. Therefore, our results should be confirmed in future large-scale investigations involving different cancer types.

Many genes have been identified as potential inducers of epithelial-to-mesenchymal transition (EMT) in the progression of CC [29,30]. *SSX2* expression was significantly higher in CC tissue samples with high disease grades than in NC tissue samples, suggesting that its expression is associated with cancer growth and metastasis. The *SSX2* gene’s role in the EMT in CC cells has not been investigated; however, the presence of *SSX2* in CC suggests it could be a therapeutic target.

Moreover, Niemeyer et al. demonstrated no *SSX1* and *SSX2* expression in patients with acute lymphatic leukemia. However, each gene was expressed in 29% of patients with acute myeloid leukemia [31]. The differences between our findings and those of the aforementioned studies may be related to the different types of leukemia samples used or the relatively small number of patients examined. Thus, additional larger-scale investigations are needed to confirm our findings.

In contrast, the expression pattern of the *SSX3* gene in the NC tissue samples was restricted to the testis, and no indication of RT-PCR expression was found in the CC tissue samples. Nonetheless, owing to the possibility of *SSX3* gene expression in other cancer types, the gene was not eliminated from the gene screening. Consequently, an RT-PCR study of this gene was performed on several types of BC, CLL, and NB tissues. This gene was found to be expressed only in the testicular tissue sample and was absent in the BC, CLL, and NB tissue samples. The study results were similar to those of a previous work, which found no evidence of *SSX3* expression in multiple human malignancies from several histological origins [16].

**Figure 10:**
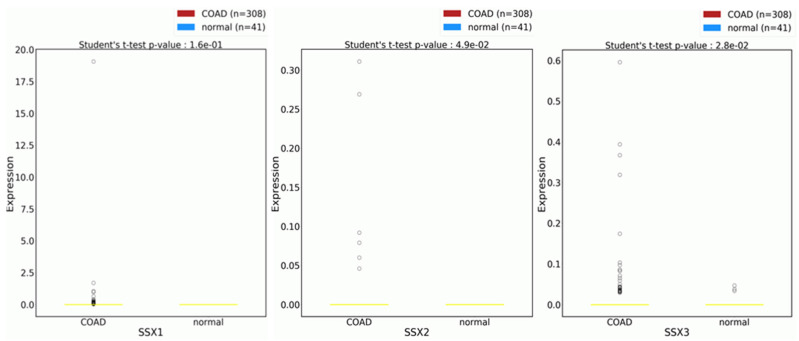
**Expression levels of the *SSX1*, *SSX2*, and *SSX3* genes in COAD and NC tissues.** The RNA sequencing data from the TCGA repository were used to create the data. *p* values ≤ 0.05 were considered significant. Abbreviations: COAD: colon adenocarcinoma; NC: normal colon.

The study examined the expression levels of the *SSX1*, *SSX2*, and *SSX3* genes in NC and colon adenocarcinoma (COAD) tissue samples using RNA sequencing data from the TCGA repository (accessed on 20 February 2023). As demonstrated by the TCGA, the expression levels of *SSX1* and *SSX2* were higher in the COAD tissue samples than in the NC tissue samples, which is consistent with our findings from the RT-PCR results in this study (Figure 10). This confirms previous research results, demonstrating increased *SSX1* and *SSX2* expression levels in numerous cancers, including CC [8,27,28,32,33]. On the other hand, the TCGA results demonstrated that the *SSX3* expression level was higher in the COAD tissue samples than in the NC tissue samples, despite the fact that both cell types expressed the gene. However, earlier research results demonstrated that *SSX3* expression was not found in several cancer types [16]. This outcome is consistent with the RT-PCR findings of the present study. Therefore, additional research is required to identify whether the TCGA results of *SSX3* are prevalent in CC tissues and the function of the *SSX3* gene in the disease.

The treatment of cancer cells with medications that deregulate DNA methylation has been demonstrated to lead to the activation of CT gene expressions in different types of cancer cells [7,11,34,35]. However, the DNA methylation regulatory mechanisms responsible for CT gene silencing have been found in only a small subset of X-CT genes, and these all are triggered by hypomethylating agents. In addition, another epigenetic mechanism that can regulate CT gene expression is the inhibition of histone deacetylation via HDACi drugs, which leads to an increase in the expression levels of different CT genes [7,11].

Epigenetic control was tested to determine whether reduced histone deacetylation or DNA methyltransferase can stimulate the expressions of the *SSX1*, *SSX2*, and *SSX3* genes. Freshly derived early-passage HCT116 and Caco-2 cell lines were treated with 100 nM of TSA or 10 µM of 5-aza-2′-CdR for 48 or 72 h, respectively. The epigenetic results demonstrated that the expression levels of *SSX1*, *SSX2*, and *SSX3* were activated with the TSA drug in the HCT116 cells but remained unaffected in the Caco-2 cells at a similar dose, which shows that not all cancer cell types react to the same treatment and maybe display tissue specificity. In addition, this observation suggests that the regulation mechanisms of *SSX1*, *SSX2*, and *SSX3* expressions may not inhibit histone deacetylation in Caco-2 cells. This observation is consistent with previous reports that indicated different expression levels of several CT genes in CC cell lines [7,11].

The greatest induction of *SSX* gene transcriptions was detected after DNA methyltransferase inhibition using 5-aza-2′-CdR. This treatment increased the expression levels of *SSX1*, *SSX2*, and *SSX3* in the HCT116 cells and those of *SSX2* and *SSX3* in the Caco-2 cells. These findings indicate that DNA hypomethylation is essential for regulating the expressions of *SSX1*, *SSX2*, and *SSX3*. It is important to study these genes as potential biomarkers and encoding therapeutic targets, and mechanistic regulatory pathways may identify categories of CT genes that are co-regulated. These results imply that several mechanisms influence the regulation of *SSX* genes. Multiple CT genes have been shown to be essential for cancer cell growth. Therefore, inactivating these genes may be advantageous for minimizing the effect of cancer and making other treatment methods more successful by reducing the proliferation-mediated burden of malignancies. DNA methylation and histone modifications have been revealed as key modulators of the EMT program. For example, CDH1 promoter methylation has been identified as an important contributor to EMT and has frequent occurrences in different human malignancies [36]. In addition, histone modifications are usually reversible and play crucial roles in defining the plasticity of EMT [37].

**Figure 11:**
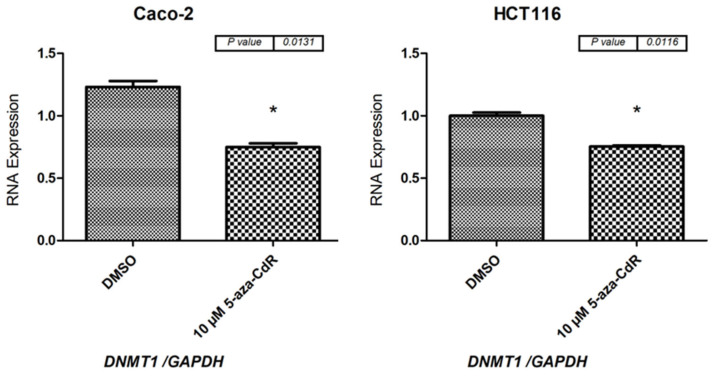
**qRT-PCR analysis of *DNMT1* expression in Caco-2 and HCT116 cells after treatment with 10 µM of 5-aza-2′-CdR for 72 h**. The bar graphs show the *DNMT1* expression levels in the Caco-2 and HCT116 cells before and after treatment with 5-aza-2′-CdR. After considering the fact that DMSO was used to dissolve the 5-aza-2′-CdR solution, this was the treatment given to the HCT116 and Caco-2 cells used as controls. GAPDH mRNA was used as a reference to normalize the expression levels. The standard error of the mean for three independent experiments is represented by the error bars. * *p* ≤ 0.05. Abbreviation: qRT-PCR: quantitative reverse transcription polymerase chain reaction.

Evidence shows that 5-aza-2′-CdR can regulate the expression of *CTCFL* (also known as *BORIS*), a transcriptional regulator that may be responsible for the regulation of numerous CT genes [38,39,40]. At this time, it is unknown whether the *SSX1*, *SSX2*, and *SSX3* gene expression alterations induced by 5-aza-2′-CdR are due to alterations in the methylation at the position of their promoter or in the expressions of other transcription factors, such as *CTCFL*, which may regulate *SSX* gene expressions. Future research should focus on elucidating the mechanism behind these changes in gene expression. In addition, from the results of this study, we raise the critical question of why induction was highly detected in the CC cell line treated with 5-aza-2′-CdR but not in the other cell lines treated with DMSO. The expression level of the primary methylation repair enzyme *DNMT1* has been reported to be decreased or degraded by 5-aza-2′-CdR treatment [41,42]. The expression level of the *DNMT1* gene decreased in HCT116 and Caco-2 cells treated with 5-aza-2′-CdR when compared to cells treated with DMSO (Figure 11). However, the role of 5-aza-2′-CdR treatment in decreasing the expression levels of other DNMT types should also be examined in future investigations.

Lastly, the aim of this research was to identify those *SSX* gene biomarkers that might aid in the screening of possible CC candidates for early detection. However, the present study has a few limitations. First, only 35 surgical samples (30 samples from male patients and five samples from female patients) were included in the study; therefore, larger samples are needed to confirm these findings. Second, the protein levels of the candidate *SSX* genes were not evaluated because of a shortage of samples.

## 5. Conclusions

The expression profiles of the three *SSX* genes were analyzed in CC and matched NC tissue samples. The gene expressions of *SSX1* and *SSX2* were detected in the CC tissue samples but not in the adjacent NC tissue samples. Therefore, these genes may be used as cancer-specific biomarkers (diagnostic tools) for the early detection of CC. However, additional protein-level investigations are needed to assess this result. This study also shows that 5-aza-2′-CdR and TSA as agents could stimulate the expressions of all *SSX* genes examined in the CC cell lines. However, on the basis of the findings of this study, we conclude that, owing to its ability to decrease *DNMT1* expression levels, 5-aza-2′-CdR is the most important regulator of *SSX* gene expression. This epigenetic regulator is important for the transcriptional activation of *SSX* genes and might be used as a therapeutic target in future cancer immunotherapies. In order to assess the effects of 5-aza-2′-CdR treatment at higher doses for longer durations or in conjunction with a TSA agent, additional research is required.

## Figures and Tables

**Table 1:** Primer sequences and sources used in the RT-PCR investigation for detecting *SSX* genes with their expected product sizes.

Primer	Accession Number	Category	Sequence (5′→3′)	Ta *	Product Size (bp)
*ACTB*	NM_001101.5	Forward Reverse	AGAAAATCTGGCACCACACC AGGAAGGAAGGCTGGAAGAG	58 °C	553
*SSX1*	NM_001278691.2	Forward Reverse	CCAGGGATGATGCTAAAGCA GTGCAGTTGTTTCCCATCGT	58 °C	389
*SSX2*	NM_175698.4	Forward Reverse	CAGAGAAGATCCAAAAGGCC CTCGTGAATCTTCTCAGAGG	58 °C	407
*SSX3*	NM_021014.4	Forward Reverse	CACGGTTGGTGCTCAAATAC TCATCTTCCTCAGGATCGCT	58 °C	528

Abbreviations: A: adenine; T: thymine; C: cytosine; G: guanine; Ta *: annealing temperature; bp: base pair.

**Table 2:** Primer sequences used in the qRT-PCR investigation for detecting *SSX* genes and their expected product sizes.

Primer	Category	Sequence (5′→3′)	Ta *	Product Size (bp)
*GAPDH*	Forward Reverse	GGGAAGCTTGTCATCAATGG GAGATGATGACCCTTTTGGC	58 °C	173
*SSX1*	Forward Reverse	ACCATAACCGCAGGATTCAG GTGCAGTTGTTTCCCATCGT	58 °C	161
*SSX2*	Forward Reverse	ACGTCCTCAGATGACTTTCG CTCGTGAATCTTCTCAGAGG	58 °C	175
*SSX3*	Forward Reverse	AACGATGGGAAACAGCTGTG TCATCTTCCTCAGGATCGCT	58 °C	161

Abbreviations: A: adenine; T: thymine; C: cytosine; G: guanine; Ta *: annealing temperature; bp: base pair.

**Table 3:** Clinical characteristics of CC, NC, BC, CLL, or NB subjects.

Variables	CC (N%)	NC (N%)	BC (N%)	CLL (N%)	NB (N%)
Participants	35 (100%)	35 (100%)	15 (100%)	12 (100%)	12 (100%)
Sex					
Males	30 (86%)	30 (86%)	-----	12 (100%)	12 (100%)
Females	5 (14%)	5 (14%)	15 (100%)	-----	-----
Mean age (min–max)	61 (24–96)	61 (24–96)	52 (32–74)	49 (39–64)	43 (33–52)
Below 61	15 (43%)	15 (43%)	-----	-----	-----
Above 61	20 (57%)	20 (57%)	-----	-----	-----
Below 52	-----	-----	9 (60%)	-----	-----
Above 52	-----	-----	6 (40%)	-----	-----
Below 49	-----	-----	-----	6 (50%)	-----
Above 49	-----	-----	-----	6 (50%)	-----
Below 43	-----	-----	-----	-----	8 (67%)
Above 43	-----	-----	-----	-----	4 (33%)
ER status					
ER+	-----	-----	6 (40%)	-----	-----
ER−	-----	-----	9 (60%)	-----	-----
PR status					
PR+	-----	-----	6 (40%)	-----	-----
PR−	-----	-----	9 (60%)	-----	-----
The cancer grades of CC patients					
Grade	I		II		III
CC patients	Males (4, 21–23, 27–29)		Males (3, 5–10, 16–20, 24–26, 30) Females (1–5)		Males (1–2, 11–15)
The cancer grades of BC patients					
Grade	I		II		III
BC patients	7, 11, 14		1–3, 6, 8, 12–13, 15		4–5, 9–10

Abbreviations: CC: colon cancer; NC: normal colon; BC: breast cancer; CLL: chronic lymphoblastic leukemia; NB: normal blood; N: number of samples; ER+: estrogen receptor positive; PR+: progesterone receptor positive.

**Table 4:** Positive *SSX1* and *SSX2* gene expressions in the NC and CC tissue samples.

*SSX* Genes	Positive Expression Number in 30 NC Samples (%)	Positive Expression Number in 30 CC Samples (%)	*p* Value
*SSX1*	0 (0%)	3 (10%)	0.078
*SSX2*	0 (0%)	6 (20%)	0.009 **

Abbreviations: NC: normal colon; CC: colon cancer. Note: values in bold represent a significant result as ** *p* ≤ 0.01.

## Data Availability

All data generated or analyzed during this study are included in this published article.
